# Causal Effects of Artificially Sweetened Foods on Chronic Pain Mediated by Gut Microbiota: A Mendelian Randomization Study

**DOI:** 10.1002/fsn3.70503

**Published:** 2025-06-22

**Authors:** Huanghong Zhao, Dongsheng Guan, Xiao Dong, Yuan Yao, Zhen Ma

**Affiliations:** ^1^ Henan Provincial Hospital of Traditional Chinese Medicine Zhengzhou China

**Keywords:** artificially sweetened foods, chronic pain, gut microbiota, mediation analysis, mendelian randomization

## Abstract

Emerging evidence suggests that both artificially sweetened foods and gut microbiota may contribute to the development and modulation of physical pain. Despite these findings, the potential mediating role of gut microbiota in the causal pathway linking artificial sweetener consumption to chronic pain remains incompletely understood. We utilized Mendelian randomization (MR) to examine the causal relationships between artificially sweetened foods, gut microbiota, and chronic pain. The data included 211 gut microbial taxa, consumption levels of nine artificially sweetened foods, and seven types of chronic pain. The primary statistical method used was inverse variance weighting (IVW). We explored whether gut microbiota mediate the relationship between artificially sweetened foods and chronic pain. We found that genetic predisposition to consuming artificially sweetened foods is associated with an increased risk of chronic pain through different types of gut microbiota. Two‐step MR suggests the mediating effects of four gut microbiota on three chronic pain: head and neck pain, joint pain, and sciatica. These findings could inform interventions and treatments for chronic pain. Artificially sweetened foods and chronic pain have causal relationships, with gut microbiota mediating the pathway from artificially sweetened food to chronic pain.

## Graphical abstract

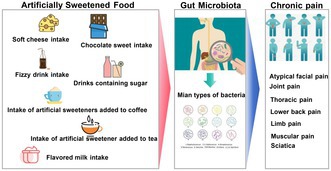

## 1 Introduction

Consuming artificially sweet foods can significantly affect human health, mainly by affecting the gut microbiota, such as altering microbial abundance, and diversity (Chi et al. 2018). Specifically, artificially sweetened foods include various sweeteners. For example, long‐term consumption of aspartame can lead to a decrease in beneficial bacteria and an increase in harmful bacteria, thereby increasing the risk of glucose intolerance and insulin resistance (Daoust 2023). Sucralose intake can reduce the number of critical anaerobic bacteria, improve gut permeability, and thus trigger inflammation and metabolic syndrome (Gómez‐Fernández et al. 2021). Saccharin may cause gut dysbiosis, which is associated with an increased incidence of glucose intolerance and type 2 diabetes. A recent clinical observational study found that long‐term aspartame consumption is related to changes in the gut microbiota, which may affect host metabolic functions (Harrington et al. 2022). Another study indicated that sucralose intake significantly reduces the number of certain beneficial gut microbiota, increasing the risk of gut permeability (Warner 2024). These studies have shown that people consuming artificially sweetened foods experience significant changes in gut microbiota, and these changes are closely linked to increased risks of metabolic disorders and chronic diseases.

The consumption of artificially sweetened foods and a preference for sweet diets can impact chronic pain through various physiological pathways (Basson et al. 2021). Artificially sweetened foods, such as aspartame and sucralose, have been found to alter the gut microbiota, thereby influencing the body's pain response mechanisms (Gerges et al. 2011). Chronic consumption of diets high in artificially sweetened foods may result in inflammation, a critical factor in chronic pain conditions (Basson et al. 2023). This inflammation can exacerbate existing pain conditions or contribute to their onset. Specifically, sweeteners like aspartame have been observed to potentially trigger headaches, a common type of chronic pain, due to their excitotoxic effects on neuronal and vascular functions (Espinosa‐Moncada et al. 2018). Therefore, while these sweetened foods are beneficial for reducing calorie intake and managing blood sugar levels, their broader impacts on health, particularly chronic pain, necessitate careful consideration and further research.

Genome‐wide association studies (GWAS) examine millions of genetic variants in an individual's genome, providing insights into complex diseases. Mendelian randomization (MR) is less affected by environmental factors and reverse causality and is a robust genetic epidemiological tool (Birney 2022). In MR studies, genetic variation is used as an instrumental variable (IV) to assess causality between exposures and outcomes and, unlike typical observational studies, this method leverages genetic variants as IV, integrating summary‐level data from large‐scale genome‐wide association studies (GWAS) to enhance statistical power and enable causal inference between exposures and outcomes (Bowden and Holmes 2019).

This study employed Mendelian randomization (MR) analysis to comprehensively investigate the causal relationship between artificially sweetened foods and chronic pain. We first established positive causal links between artificially sweetened foods, gut microbiota, and chronic pain, supported by robust sensitivity analyses. Subsequently, we explored whether gut microbiota mediates the pathway from artificially sweetened foods to chronic pain, contrasting our genetically informed findings with those from observational studies. By elucidating this mediating role, our research provides valuable insights into the impact of dietary habits on chronic pain and has significant clinical implications.

## 2 Methods

### 2.1 Study Design

**FIGURE 1:**
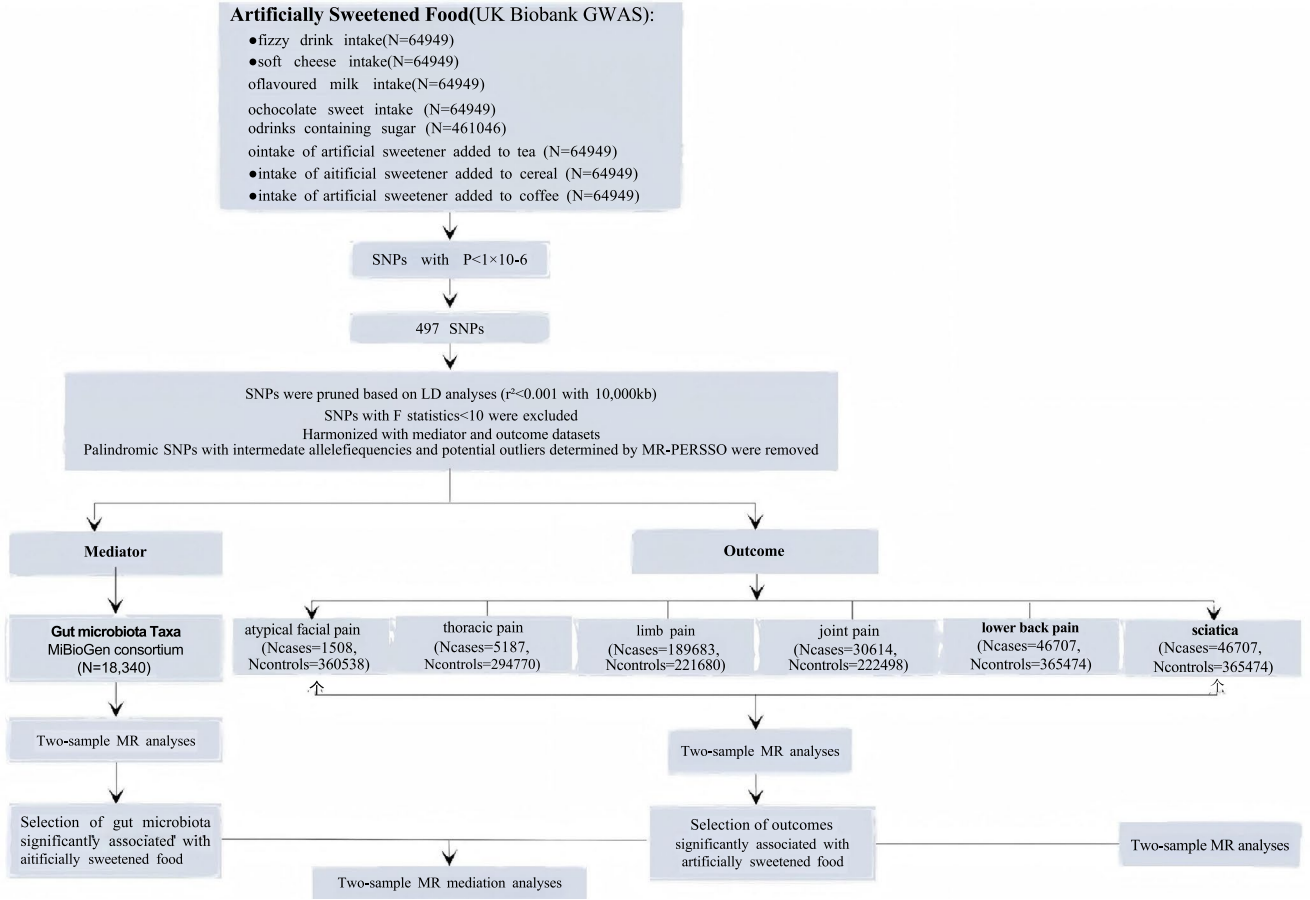
The flowchart illustrating the design of the study.

Our study provided a comprehensive flowchart illustrating the design of the study in detail (Figure 1). First, we performed two‐sample analyses based on a recent large‐scale genome‐wide association study (GWAS) of individuals of European ancestry, followed by a two‐step magnetic resonance approach to assess potential relationships between artificially sweetened foods and chronic pain, and evaluate the potential gut microbiota as the mediations in this relationships. This paper is a secondary analysis of publicly available summaries of GWAS data analyses. Ethical approval was obtained for each of the original GWAS studies, and no individual‐level data were used in this investigation, so no further ethical review board consent was required.

### 2.2 Data Source

Genetic data on the gut microbiota were obtained from the MiBioGen consortium, an analysis spanning 24 cohorts, including genome‐wide genotypes and 16S fecal microbiome data for 18,340 individuals. The GWAS summary data included 211 gut microbial taxa (131 genera, 35 families, 20 orders, 16 classes, and 9 species) (Kurilshikov et al. 2021).

Our study leverages comprehensive phenotype data about artificially sweetened foods from the UK Biobank (https://www.ukbiobank.ac.uk). This rich dataset encompasses detailed information on chocolate sweet intake, soft cheese intake, flavored milk intake, fizzy drink intake, drinks containing sugar, and artificial sweeteners added to cereal, coffee, and tea. In the assessment of food exposure data, the selection criteria for soft cheese required: (a) industrial reprocessing with ≥ 10% animal‐derived ingredients and (b) addition of ≥ 5 g/L artificial sweeteners (e.g., aspartame, sucralose), with data sourced from the EFSA FoodEx2 database and dairy production records across 12 EU member states (2015–2020), while excluding traditional non‐sweetened cheeses (Iizuka 2022). For drinks containing sugar, compliance with EU Regulation (EU) No 1333/2008 mandated exclusive use of non‐nutritive sweeteners, validated via HPLC to confirm concentrations ≥ 1 g/L in industrialized drinks, with systematic exclusion of naturally sugary beverages (e.g., fruit juices) (Jones et al. 2023).

Chronic pain, a persistent or recurring painful sensation lasting more than 3 months (Von Korff et al. 2020), was assessed using data obtained from the 8th version of the FinnGen consortium (https://r8.risteys.fnngen.fi/). This dataset includes information on atypical facial pain, thoracic pain, limb pain, joint pain, lower back pain, and sciatica. The FinnGen study adheres strictly to the International Classification of Headache Disorders (ICHD‐3) and IASP chronic pain criteria for diagnosing pain phenotypes (Moon et al. 2019; Schweiger et al. 2025). For example, the diagnostic criteria for atypical facial pain include: ① Localization conforming to the trigeminal nerve distribution (V1–V3), excluding episodic electric shock‐like pain (ICHD‐3 code 13.1.2); ② Duration exceeding 4 h per day with a disease course ≥ 3 months; and ③ Exclusion of other potential causes through head MRI (excluding tumors/multiple sclerosis), dental panoramic X‐ray (excluding dental caries), and serum anti‐SSA/SSB testing.

### 2.3 Instrumental Variables Selection

We set the threshold for significant association between single nucleotide polymorphisms (SNPs) and artificially sweetened foods to *p* < 5 × 10^−6^. Similarly, for screening SNPs related to gut microbiota, a relatively relaxed *p* < 5 × 10^−6^ threshold was used to utilize existing genetic tools fully. We excluded any SNPs that exhibited linkage disequilibrium (LD) with an LD cutoff of *r* ^2^ < 0.001 and isolated by more than 10,000 kb from our analysis. Furthermore, we removed SNPs that met these criteria to ensure robustness in our findings. In the Mendelian randomization (MR) framework, it is essential to ensure that the effects of SNPs on exposure align with the impacts of associated alleles on the outcomes (Widding‐Havneraas and Zachrisson 2022). To maintain data integrity, alleles were removed post‐matching.

### 2.4 Primary Analysis

We conducted two‐sample Mendelian randomization (MR) analyses to assess the causal effects of artificially sweetened foods and gut microbiota on chronic pain. For exposures with multiple instrumental variables (IVs), the inverse‐variance weighted (IVW) method served as the primary analytical approach. In cases where only a single IV was available, causal estimates were derived using the Wald ratio. All MR estimates are reported with 95% confidence intervals (CIs) and odds ratios (ORs). Statistical significance was defined as IVW *p* < 0.05, with concordant directional estimates between IVW and MR‐Egger analyses strengthening result robustness (Zhang and Ghosh 2017).

### 2.5 Bidirectional Causality Analysis

We consider chronic pain as a form of “exposure” and consider the possible consumption of artificially sweet foods by pain patients as a “result” to evaluate whether there is a bidirectional causal relationship between artificially sweet foods and chronic pain. As IVs, we chose SNPs closely related to chronic pain (*p* < 5 × 10^−6^).

### 2.6 Mediation Analysis

We conducted two‐sample Mendelian randomization to assess the mediating role of gut microbiota in the pathway from artificially sweetened foods (ASFs) to chronic pain. For significant ASF‐pain associations (*p* < 0.05), candidate mediators were selected based on the following criteria: (1) microbial taxa causally affected by ASFs (*p* < 5 × 10^−6^); and (2) taxa demonstrating causal effects on pain outcomes (*p* < 0.05/211).

The mediation proportion was calculated using the product method:

$$\text{Mediation Proportion}=\frac{\beta_{\mathrm{ASF}\to \text{mediator}}\times \beta_{\text{mediator}\to \text{pain}}}{\beta_{\text{total effect}\;\left(\mathrm{ASF}\to \text{pain}\right)}}$$

### 2.7 Sensitivity Analysis

All statistical analyses were conducted using R (version 4.3.2). Mendelian randomization (MR) analyses were performed with the TwoSampleMR package (version 0.5.7), with Cochran's *Q* test applied to evaluate SNP heterogeneity (Gurung et al. 2020; Plana et al. 2021). Scatter plots visualizing SNP‐exposure‐outcome associations were generated to illustrate MR effect estimates (Bowden and Holmes 2019).

## 3 Results

### 3.1 Genetic Instruments

The selection of instrumental variables (IVs) employed exposure‐specific genome‐wide significance thresholds and quality control protocols. For gut microbiota, genome‐wide significant SNPs (*p* < 5 × 10^−6^) from the MiBioGen consortium underwent linkage disequilibrium (LD) clumping (*r* ^2^ < 0.001, 10,000 kb window; PLINK v2.0), yielding 2560 independent IVs across 211 microbial taxa (mean 12.1 SNPs/taxon; range 3–29; Table S1). For artificially sweetened foods (ASFs), a stricter threshold (*p* < 5 × 10^−6^) was applied to UK Biobank data with identical LD clumping parameters, identifying 497 IVs (Table S2). All IVs demonstrated robust instrument strength, with *F*‐statistics exceeding 10 (mean *F* = 23.4; min *F* = 10.3), effectively mitigating weak instrument bias.

### 3.2 The Causal Effects of Intake of Artificially Sweetened Foods on Chronic Pain

**FIGURE 2:**
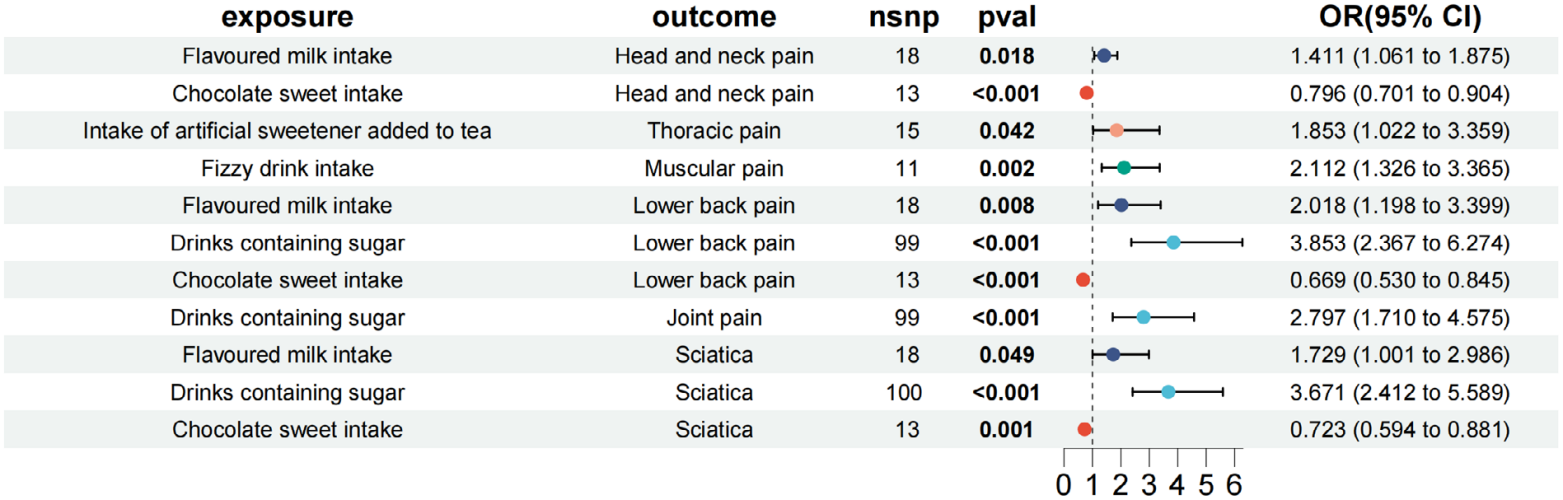
The causal effects of intake of artificially sweetened foods on chronic pain.

As illustrated in Figure 2, flavored milk consumption was associated with an increased likelihood of head and neck pain (OR = 1.411, 95% CI = 1.061 to 1.875, *p* = 0.018), while artificial sweeteners in tea correlated with a higher incidence of thoracic pain (OR = 1.853, 95% CI = 1.022 to 3.359, *p* = 0.042). Consumption of fizzy drinks was linked to muscular pain (OR = 2.112, 95% CI = 1.326 to 3.365, *p* = 0.002). Additionally, flavored milk intake was associated with increased occurrence of low back pain (OR = 2.018, 95% CI = 1.198 to 3.399, *p* = 0.008), as were drinks containing artificial sugar (OR = 3.835, 95% CI = 2.367 to 6.274, *p* < 0.001). Drinks containing artificial sugar also correlated with a higher risk of joint pain (OR = 1.033, 95% CI = 1.007 to 1.060, *p* = 0.014). Furthermore, flavored milk intake (OR = 1.729, 95% CI = 1.001 to 2.986, *p* = 0.049) and drinks containing sugar (OR = 3.671, 95% CI = 2.412 to 5.589, *p* < 0.001) were associated with increased risk of sciatica.

### 3.3 The Causal Effects of Chronic Pain on Intake of Artificially Sweetened Foods

As illustrated in Table S3, MR analysis reveals that limb pain (OR = 1.11, 95% CI = 1.014 to 1.281, *p* = 0.032) and low back pain (OR = 1.213, 95% CI = 1.107 to 1.196, *p* = 0.004) were associated with drinks containing sugar. Muscular pain (OR = 1.051, 95% CI = 1.011 to 1.063, *p <* 0.001) and limb pain (OR = 1.061, 95% CI = 1.028 to 1.111, *p* = 0.042) were associated with chocolate sweet intake. Limb pain (OR = 1.057, 95% CI = 1.038 to 1.158, *p <* 0.01), joint pain (OR = 1.032, 95% CI = 1.023 to 1.067, *p* = 0.014), and low back pain (OR = 1.033, 95% CI = 1.035 to 1.061, *p* = 0.012) were associated with the consumption of fizzy drinks.

### 3.4 The Causal Effects of Intake of Artificially Sweetened Foods on Gut Microbiotas

**FIGURE 3:**
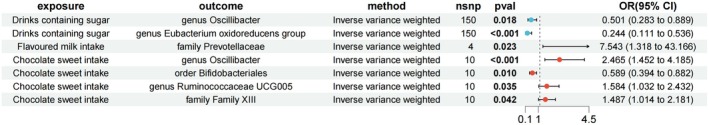
The causal effects of intake of artificially sweetened foods on gut microbiotas.

As illustrated in Figure 3, MR analysis screened a total of 211 gut microbiotas for a causal relationship with artificially sweetened foods (*p* < 0.05), in which seven gut microbiotas were most likely to be involved in the physiological processes that lead to chronic pain on an artificially sweetened diet, that is drinks containing sugar could reduce the abundance of genus Oscillibacter (OR = 1.127, 95% CI = 1.094 to 1.161, *p* = 0.018) and genus Eubacterium oxidoreductase group (OR = 0.244, 95% CI = 0.111 to 0.536, *p* < 0.001). Chocolate sweet intake could reduce the abundance of order Bifidobacteriales (OR = 0.589, 95% CI = 0.394 to 0.882, *p* = 0.010). However, flavored milk intake could increase the abundance of the family Prevotellaceae (OR = 7.543, 95% CI = 1.318 to 43.166, *p* = 0.023). The increased abundance of genus Oscillibacter (OR = 2.465, 95% CI = 1.452 to 4.185, *p* < 0.001), genus Ruminococcaceae UCG005 (OR = 1.584, 95% CI = 1.032 to 2.432, *p* = 0.035), and family Family XIII (OR = 1.487, 95% CI = 1.014 to 2.182, *p* = 0.042), were all associated with chocolate sweet intake.

### 3.5 The Causal Effects of Gut Microbiotas on Chronic Pain

**FIGURE 4:**
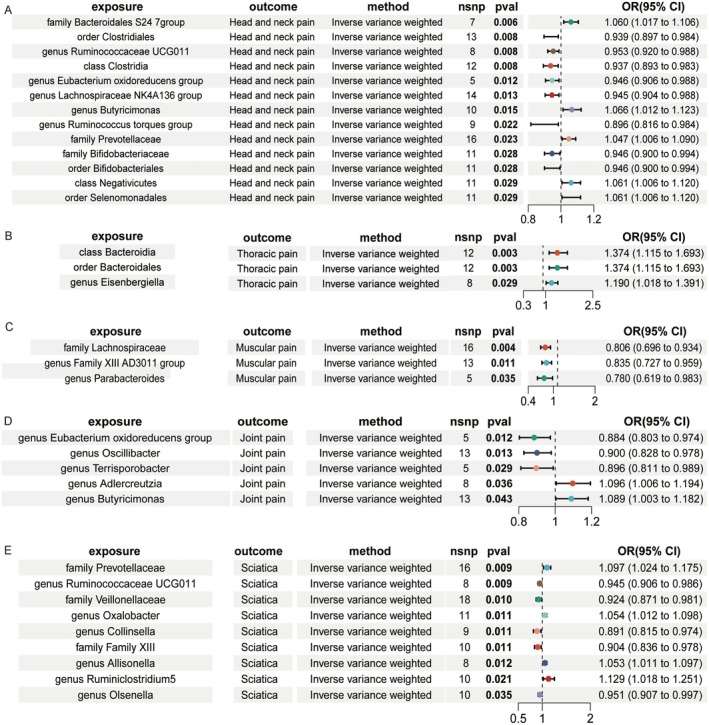
The causal effects of gut microbiotas on chronic pain.

As illustrated in Figure 4, Based on the impact of artificially sweetened foods on the gut microbiota mentioned above, we can examine the effect of the same microbiota taxa on different pain properties. MR analysis reveals that the genus Eubacterium oxidoreductase group (OR = 0.946, 95% CI = 0.906 to 0.988, *p* = 0.012), the order Bifidobacteriales (OR = 0.946, 95% CI = 0.900 to 0.994, *p* = 0.028), and the family Prevotellaceae (OR = 1.047, 95% CI = 1.006 to 1.090, *p* = 0.028) were associated with lower risk of head and neck pain. The genus Oscillibacter (OR = 0.900, 95% CI = 0.828 to 0.978, *p* = 0.013) was associated with a lower risk of joint pain. The family Family XIII (OR = 0.904, 95% CI = 0.836 to 0.978, *p* = 0.011) was associated with a lower risk of sciatica.

### 3.6 Mediation Analysis

Using the two‐step Mendelian randomization (MR) method for mediation analysis, we present in Table 1 the outcomes and the proportion of mediation attributed to specific factors. Our findings show that the genus Oscillibacter significantly mediates 42.8% of the relationship between drinks containing sugar and joint pain. The family Prevotellaceae mediates 13.8% of the relationship between s flavored milk intake and head and neck pain. The order Bifidobacteriales mediates 13% of the relationship between sweet chocolate intake and head and neck pain. The family Family XIII mediates 2.1% of the relationship between sweet chocolate intake and sciatica.

**TABLE 1:** Mendelian randomization analyses of the causal effects between Artificially sweetened food, gut microbiota, and chronic pain.

Exposure	Outcome	Mediator	Mediation effect			
			Direct effect (*β*1* ± SE)	Indirect effect (ɑ* ± SE)	Indirect effect (*β*2* ± SE)	Proportion mediated (*ɑ* × *β*2*/*β*1)
Chocolate sweet intake	Head and neck pain	Order Bifidobacteriales	−0.228 ± 0.065	−0.529 ± 0.206	0.056 ± 0.025	0.13
Flavored milk intake	Head and neck pain	Family Prevotellaceae	0.344 ± 0.145	2.021 ± 0.890	0.046 ± 0.020	0.27
Drinks containing sugar	Joint pain	Genus Oscillibacter	0. 171 ± 0.080	−0.691 ± 0.292	−0. 106 ± 0.043	0.428
Chocolate sweet intake	Sciatica	Family Family XIII	0.49 ± 0.075	−0. 101 ± 0.195	−0. 101 ± 0.04	0.021

*Note:* Beta (*β*), standard errors (SE), and *p*‐values were obtained from multivariable Mendelian randomization analysis. *β*1* and *β*2* represent the controlled direct effects of each pair of artificially sweetened food and gut microbiota on chronic pain after adjusting for each other. *α* is the causal effect of exposure on mediator; indirect effect (*α* × *β*2*) is the effect of exposure on chronic pain via corresponding mediator; *β*1 is the total effect of exposure on chronic pain; proportion mediated is calculated as the “indirect effect/total effect.”

## 4 Discussion

Our study takes a novel approach, using a comprehensive Mendelian randomization (MR) investigation to uncover the intricate relationships between artificial sweetener foods and chronic pain. We utilized large‐scale summary‐level statistics from GWAS and discovered that genetic susceptibility to artificial sweetener foods was linked to an increased risk of chronic pain. Meanwhile, we identified the involvement of gut flora in the pathology of sweeteners affecting chronic pain, including head and neck pain, joint pain and sciatica.

The phenotype of chronic pain in specific body areas may involve complex and multifaceted mechanisms, encompassing factors, such as genetic predisposition, lifestyle choices, and other unmeasurable variables (Mak and Schneider 2020). Inter‐individual differences are among the most crucial factors (Edwards 2005). Due to the unpredictability and unquantifiability of pain triggers, as well as the need for more objective evidence in clinical settings regarding individual differences leading to chronic pain, there still needs to be a gap in understanding (Mogil 2021). Little research has been conducted to elucidate whether artificially sweetened food serves as a precursor to chronic pain episodes (Patel et al. 2006). Therefore, our study aims to contribute objective clinical evidence by exploring whether artificially sweetened food can serve as prodromes for chronic pain episodes.

We screened nine artificially sweetened foods to verify that sweetener intake increases the risk of chronic pain. A study that followed the dietary intake of more than 20,000 participants for 9 years found that people who consumed more than one serving of artificial sweeteners daily had more than twice the risk of chronic headaches compared to those who did not use artificial sweeteners (Newman and Lipton 2001). In clinical practice, in response to the potential for artificial sweeteners to adversely affect the nervous system, some physicians have begun to advocate reducing the intake of artificial sweeteners, such as discontinuing aspartame‐containing beverages, reducing cheese intake, and limiting the intake of flavored yogurt, as an essential part of treating or preventing pain (Guo et al. 2019). Our findings further strengthen the evidence that genetically predicted sweetener diets are positively associated with chronic pain risk. Interestingly, this study found a high odds ratio (OR = 3.835) between sugary drinks and lower back pain. This finding is potentially influenced by cumulative lifetime exposure and sample overlap bias, factors that may conflict with previously observed data. Therefore, the odds ratio should be interpreted with caution. However, the underlying biological rationale supports a causal relationship.

In an intermediate mediator analysis, we further quantified the mediating role of gut microbes in the relationship between artificially sweetened foods and chronic pain. Five of the nine sweetened foods in the study mediated three potential pain categories (e.g., head and neck pain, joint pain, and sciatica) through the gut microbes. Recent studies have shown that interactions between different microbial species in the gut, on the one hand, can alter the intestinal microenvironment and influence neuronal excitability and pain perception. For example, interactions between Gram‐negative and Gram‐positive bacteria can lead to the expression of pain‐associated sensory receptors in the host's gut (Shen et al. 2017); on the other hand, dysbiosis of the gut microbiota can disrupt the intestinal barrier and increase endotoxin permeability. For example, patients with chronic inflammatory bowel disease (IBD) exhibit microbial imbalances and a compromised intestinal mucosal barrier, which is associated with their chronic pain (Li et al. 2022). In addition, microbial communities modulate the host's nervous and immune systems, influencing gut sensory transduction and pain sensitivity. For example, anaerobic bacteria such as Enterococcus can modulate neuronal activity in the gut by releasing short‐chain fatty acids, which enhance pain signaling (Defaye et al. 2020). Specific gut microbiota can release inflammatory mediators, such as tumor necrosis factor (TNF‐ɑ), and interleukin (IL‐6) from host immune cells. These molecules are directly involved in the neuroinflammatory response in the gut, which enhances pain perception (Bi et al. 2024).

Experimental studies demonstrate that Oscillibacter‐derived butyrate deficiency in IBS patients correlates with NMDA receptor‐mediated central sensitization, while *Prevotella copri* elevates serum kynurenine to activate micro (Li et al. 2024). Preclinically, *Faecalibacterium prausnitzii* increases hippocampal BDNF via butyrate‐induced HDAC inhibition (Ma et al. 2020), reversing neuropathic hyperalgesia, and *Lactobacillus reuteri* suppresses visceral pain through histamine‐dependent vagal inhibition of macrophage TNF‐ɑ (Deng et al. 2024). These pathways align with our findings, suggesting that sweeteners may dysregulate pain by altering key taxa (e.g., depleting butyrate producers) and their effector metabolites (SCFAs, tryptophan derivatives). The convergence of clinical biomarker data and animal model evidence substantiates a causal microbiota‐pain axis mediated by gut‐brain crosstalk.

Preliminary findings suggest a causal relationship between artificially sweetened foods and chronic pain, with important implications for public health and clinical practice (Lin et al. 2020). The results provide a rationale for interventions to prevent or treat these conditions. Given the observed link between artificial sweeteners and chronic pain, interventions targeting artificial sweeteners may serve as a means of avoiding or controlling pain (Patel et al. 2006). Treating poor dietary habits, such as artificial sweeteners, as essential factors in early screening for chronic pain could be effective in improving chronic pain prevention (Newman and Lipton 2001). Promoting a healthy lifestyle and reducing the intake of artificially sweetened foods are of great value in relieving chronic pain (Ding et al. 2021).

The main strength of our current study lies in carefully using a two‐way Mendelian randomization design in both samples, effectively employing three Mendelian randomization (MR) methods (IVW, MR‐Egger, and Weighted Median) to reduce bias and confounding (Park 2020). This study employed SNP thresholds based on exposure category. ① For nine categories of artificially sweetened foods, given the limited GWAS sample size and to avoid insufficient instrumental variables (IVs), the threshold was relaxed to *p* < 5 × 10^−6^. This ensured an adequate number of IVs while maintaining sufficient instrument strength. ② For gut microbiota that exhibit significant polygenicity due to GWAS and whose SNPs are typically located in host‐microbe co‐evolutionary regions (such as HLA loci), a relatively relaxed threshold of *p* < 5 × 10^−6^ should be applied to explore the potential mediating role of the microbiota as much as possible. The validity of both exposure types was confirmed using the MR‐Egger intercept test and the weighted median method, demonstrating that the robustness of the results was not compromised by weak instruments or confounding bias (Blanchini et al. 2022).

While our study is pioneering in nature, it is important to acknowledge its limitations. Despite our efforts to avoid sample overlap by using different public databases from Finland, UKB, and IEUopen GWAS, the inability to access detailed participant information to eliminate potential sample overlap from vast databases could potentially impact the robustness of our Mendelian randomization (MR) results. Our investigation is also confined solely to the European population, which limits the generalizability of our findings to other demographic cohorts; therefore, cross‐racial validation is necessary. Additionally, while our focus is on gut microbiota as a mediator between artificial sweetener foods and chronic pain, there may be other potential confounding factors. These variables, possibly due to unmeasured or confounded variables, may affect our analysis given the inherent complexity and potential limitations highlighted. Therefore, further research with more diverse populations and comprehensive datasets must be undertaken to validate and extend our findings.

## 5 Conclusion

In this study, we comprehensively explored whether artificial sweetener foods and gut microbiotas significantly impact the development of chronic pain. Initially, we clarified the positive causal relationships between artificial sweetener foods, gut microbiotas, and chronic pain and conducted sensitivity analyses to support our findings. We identified three vital artificial sweetener foods that intricately regulate the progression of three types of pain. Chocolate sweet intake and flavored milk intake are closely associated with increased head and neck pain risk. Drinks containing sugar have a positive correlation with the risk of joint pain, and sweet chocolate intake explicitly drives an increased risk of sciatica. The findings highlight a causal relationship between artificial sweetener food and chronic pain, with gut microbiotas mediating this relationship.

## Author Contributions

**Huanghong Zhao:** data curation (equal), software (supporting). **Dongsheng Guan:** formal analysis (lead), validation (lead). **Xiao Dong:** methodology (equal). **Yuan Yao:** project administration (equal). **Zhen Ma:** formal analysis (equal), supervision (equal).

## Ethics Statement

The present study is a secondary analysis of publicly available data. Ethical approval was granted for each of the original GWAS studies. In addition, no individual‐level data were used in this study. Therefore, no new ethical review board approval was required.

## Consent

The authors have nothing to report.

## Conflicts of Interest

The authors declare no conflicts of interest.

## Supporting information


**Table S1.** Instrumental variables for gut microbiotas.
**Table S2.** Instrumental variables fo artificially sweetened food consumption.
**Table S3.** The causal effects of Chronic pain on intake of artificially sweetened foods.

## Data Availability

All data used in the present study were obtained from genomewide association study summary statistics which were publicly released by genetic consortia.
