# Supplementary material for: Causal Effects of Artificially Sweetened Foods on Chronic Pain Mediated by Gut Microbiota: A Mendelian Randomization Study
Source: Food Sci Nutr. 2025 Jun 22;13(6):e70503. doi: 10.1002/fsn3.70503 (PMC12183110; doi:10.1002/fsn3.70503)
Supplement: Supplementary file 1 — Table S1. Instrumental variables for gut microbiotas. Table S2. Instrumental variables fo artificially sweetened food consumption. Table S3. The causal effects of Chronic pain on intake of artificially sweetened foods. [file FSN3-13-e70503-s001.zip › Supplementary chart information.docx]

**Supplementary chart information**

**Table S1.** Instrumental Variables for Gut microbiotas .

**Table S2:** Instrumental Variables fo Artificially Sweetened Food Consumption.

**Table S3:**The causal effects of Chronic pain on intake of Artificially sweetened foods.
